# Graphdiyne as a promising material for detecting amino acids

**DOI:** 10.1038/srep16720

**Published:** 2015-11-16

**Authors:** Xi Chen, Pengfei Gao, Lei Guo, Shengli Zhang

**Affiliations:** 1 Department of Applied Physics, School of Science, Xi’an Jiaotong University, Xi’an, China

## Abstract

The adsorption of glycine, glutamic acid, histidine and phenylalanine on single-layer graphdiyne/ graphene is investigated by *ab initio* calculations. The results show that for each amino acid molecule, the adsorption energy on graphdiyne is larger than the adsorption energy on graphene and dispersion interactions predominate in the adsorption. Molecular dynamics simulations reveal that at room temperature the amino acid molecules keep migrating and rotating on graphdiyne surface and induce fluctuation in graphdiyne bandgap. Additionally, the photon absorption spectra of graphdiyne-amino-acid systems are investigated. We uncover that the presence of amino acid molecules makes the photon absorption peaks of graphdiyne significantly depressed and shifted. Finally, quantum electronic transport properties of graphdiyne-amino-acid systems are compared with the transport properties of pure graphdiyne. We reveal that the amino acid molecules induce distinct changes in the electronic conductivity of graphdiyne. The results in this paper reveal that graphdiyne is a promising two-dimensional material for sensitively detecting amino acids and may potentially be used in biosensors.

The interaction between biological molecules and materials is a significant topic in condensed matter physics and material science research. In designing bio-devices, especially nano biosensors, a fundamental problem is to explore the physical mechanism of the interactions between amino acids (AAs) or other biological molecules and material surfaces. Recent progress in understanding the physical-chemical processes occurring in bio-inorganic interfaces has led to major developments in biomedicine and other corresponding areas^1^,^2^,^3^,^4^,^5^.

The remarkable success in preparing graphene (GP)^6^,^7^,^8^,^9^, molybdenum disulfide^10^,^11^,^12^ and other two-dimensional materials^13^,^14^,^15^ provides more possibilities for developing sensitive bio-devices or medicine systems. GP, a promising material for various applications in engineering and medicine, is considered a flexible substrate that can be functionalized with peptides, proteins and small biomolecules^16^,^17^. A detailed understanding of the interactions between proteins and GP may facilitate the development of advanced biological applications such as biosensors for the detection of biomolecules^18^,^19^,^20^,^21^, living cells^22^,^23^, drug delivery systems^24^ and cell imaging. However, as a semi-metal with zero bandgap^7^,^25^,^26^, GP is limited in sensitive electrical detection applications for biomolecules or other cases. Fortunately, graphyne and its family (namely graphdiyne, graphyne-3, graphyne-4 *etc*.) are theoretically predicted as two-dimensional semiconducting C allotropes^27^,^28^ and have the potential to replace GP in electronic applications. Recently, graphdiyne (GD) was successfully synthesized on the surface of copper via a cross-coupling reaction using hexaethynylbenzene^29^. As an intrinsic semiconducting C material with a bandgap, GD is more suitable than GP for fabricating nanoelectronic devices. Besides, compared with GP, GD has a unique structure of larger pores and these pores are composed of high π-conjugated acetylenic bonds, which may have strong adsorption to biomolecules. In our previous work, the electronic transport properties of GD have been theoretically investigated^30^. For fabricating GD-based bio-devices, the interaction between GD and AA molecules and the influence of AA molecules on the electronic and optical properties of GD should be further investigated.

In this work, the interactions between single-layer GD and typical AAs (glycine (Gly), glutamic acid (Glu), histidine (His) and phenylalanine (Phe)) are investigated theoretically by *ab initio* calculations and compared with the interactions between single-layer GP and AAs. According to the classification of AAs, we choose Gly as a typical nonpolar aliphatic AA, Phe as a typical aromatic AA, His as a typical positively charged AA and Glu as a typical negatively charged AA. Firstly, molecular dynamics (MD) simulations are employed for probing the thermal motions of AA molecules on GD surface and searching the most stable configurations of AA molecules adsorbed on GD. According to the results, the adsorption energy of each AA molecule on GD is found larger than the adsorption energy of the AA molecule on GP, leading us to investigate the influence of AA molecules on the bandgap and photon absorption spectrum of GD. We find that the adsorbed AA molecules induce fluctuation in GD bandgap, while the photon absorption spectrum of GD is depressed and shifted by the AA molecules. Finally, quantum electronic transport simulations are performed for the GD-AA systems. The current-bias curves of GD-AA systems are compared with the current-bias curve of pure GD, displaying the response of GD to different AAs. The above results indicate that GD is a promising two-dimensional material for sensitive AA/protein biosensors, and this work should be beneficial to the future design of GD-based AA/protein biosensors, GD-based drug delivery or other GD-based nano biological devices.

## Results

### Structure of GD and AAs

**Figure 1:**
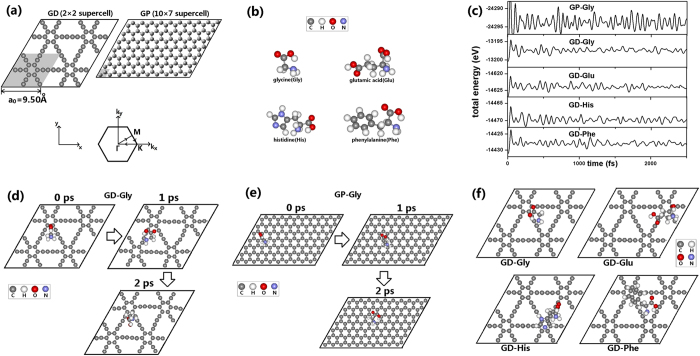
(a) The (2 × 2)/(10 × 7) hexagonal supercell of the GD/GP layer and their Brillouin zone. The unit cell of GD/GP is presented by the gray area. The lattice constant *a*_0_ of GD is 9.50 Å. (**b**) The structures of AA molecules. (**c**) The total energy profile in the MD simulations. (**d**) The snapshots of GD-Gly system at 300 K. (**e**) The snapshots of GP-Gly system at 300 K. (**f**) The most stable configurations of GD-Gly, GD-Glu, GD-His and GD-Phe.

Figure 1(a) presents the structure of GD, with the unit cell shown by the gray area. GD is composed by the hexagonal rings of *sp*^2^ C atoms connected by 4-atom *sp* C-C chains. The C-C bonds in the 4-atom chains present alternating single and triple bonds. Our optimized lattice constant *a*_0_ = 9.50 Å (Fig. 1(a)) is in good agreement with the value of 9.48 Å calculated using the projector-augmented-wave method^31^.

Since AAs are classified as non-polar aliphatic, polar aliphatic, non-polar aromatic and polar aromatic, in the following simulations we choose Gly as a typical non-polar aliphatic AA, Glu as a typical polar aliphatic AA, His as a typical polar aromatic AA and Phe as a typical non-polar aromatic AA. The optimized structures of free AAs are shown in Fig. 1(b).

### Thermal motion of AAs on GD/GP

To investigate the thermal motion of AA molecules on GD/GP, *ab initio* MD simulations were employed using a (2 × 2)/(10 × 7) hexagonal supercell (Fig. 1(a)). In the beginning, an AA molecule was put on the surface of GD/GP sheet with a random initial position and orientation. The geometries of these GD-AA/GP-AA systems were optimized. Then, constant-temperature MD simulations were performed at 300 K to explore whether the system could remain stable. During the simulations, the energy-time profile is evidence for judging whether the system is in equilibrium. For example, for the GP-Gly system at 300 K, the total energy fluctuates with time in a range of about 5 eV (the 1^st^ panel of Fig. 1(c)). The GP-Gly system contains 150 atoms and thus the energy fluctuation per atom is about 0.03 eV, close to the average atomic translational energy 3*kT*/2. This result indicates that the GP-Gly system is in equilibrium. Similarly, the GD-Gly, GD-Glu, GD-His and GD-Phe systems (the 2^nd^, 3^rd^, 4^th^, and 5^th^ panel of Fig. 1(e), respectively) contains 82, 91, 92 and 95 atoms, respectively. Their total energy fluctuations all keep a range of about 3 eV, with their energy fluctuation per atom about 0.03 eV, also getting close to 3*kT*/2. The above results indicate that the GD-AA/GP-AA systems are all in equilibrium at 300 K.

Our MD simulations show that AA molecules constantly migrate and rotate on the GD/GP surface. For example, Fig. 1(d) presents some snapshots of GD-Gly at 300 K. The Gly molecule randomly moves on the GD surface and continuously changes its position and orientation. During the MD simulation, the Gly molecule maintains a distance of about 2~3 Å from the GD surface. For the GP-Gly system, we can also see the random walk of Gly on the GP surface (Fig. 1(e)), where the Gly molecule also maintains a distance of about 2~3 Å from the GP surface. Actually, for the GD-Glu/GP-Glu, GD-His/GP-His and GD-Phe/GP-Phe systems at 300 K, it can be observed that all of the AA molecules constantly migrate and rotate on the GD/GP surface, with the distance of 2~3 Å from the GD/GP surface. We also performed MD simulations at 800 K and the AA molecules are still confined to the GD/GP surface with a distance of 3~4 Å. The above results indicate that the GD-AA/GP-AA systems are thermally stable.

### Adsorption of AAs on GD/GP

To investigate the adsorption energy *E*_ad_ of AA on GD/GP, some configurations with low potential energy were chosen from the above MD simulations to perform geometry optimizations. Using this procedure, we obtained a series of stable structures of GD-AA/GP-AA with minimum total energies. Among these stable structures, the most stable configuration with the largest adsorption energy *E*_ad_ can be found. In the above procedure, the *E*_ad_ was calculated using the Perdew-Burke-Ernzerhof (PBE) functional^32^, DFT-D2 empirical dispersion correction for PBE (PBE-D2)^33^ and non-local van der Waals functional (vdW-DF)^34^. For GD-Gly, GD-Glu, GD-His and GD-Phe, the most stable structures found by the above procedure are presented in Fig. 1(f). For all the GD-AA/GP-AA systems, the values of *E*_ad_ are listed in Table 1. The adsorption of AA on GD/GP should be stable because the value of *E*_ad_ is much larger than the molecular translational kinetic energy (~3*kT*/2). For GD-Gly, the largest adsorption energy *E*_ad_ calculated using the PBE/PBE-D2/vdW-DF functional are 0.59/0.90/1.10 eV, respectively. For GP-Gly, the largest adsorption energy *E*_ad_ calculated using the PBE/PBE-D2/vdW-DF functional was found to be 0.23/0.53/0.54 eV, respectively. It is worth noting that our calculated *E*_ad_ of GP-Gly at the level of PBE is in agreement with previous *ab initio* calculations^35^,^36^,^37^. *E*_ad_ for GD-Glu is 0.54/0.90/1.14 eV using PBE/PBE-D2/vdW-DF, respectively. *E*_ad_ for GD-His is 0.73/1.22/1.46 eV using PBE/PBE-D2/vdW-DF, respectively. *E*_ad_ for GD-Phe is 0.77/1.27/1.53 eV using PBE/PBE-D2/vdW-DF, respectively. Additionally, we note that the *E*_ad_ of GP-His and GP-Phe calculated using the second-order Møller–Plesset perturbation theory^38^ is smaller than the PBE-D2/vdW-DF value and larger than the PBE value. The above calculations (Table 1) indicate that the *E*_ad_ of Gly on GD is larger than the *E*_ad_ on GP. For Glu, His and Phe, we also compared their *E*_ad_ on GD with the *E*_ad_ on GP. Actually, for every AA we found that the *E*_ad_ on GD are all larger than the *E*_ad_ on GP (Table 1). This is potentially caused by the acetylenic bonds in GD which have more π-electrons than the ethylenic bonds in GP and causes stronger bonding to AA molecules.

According to the above results, for all GD-AA/GP-AA systems, *E*_ad_ calculated using PBE is obviously smaller than *E*_ad_ calculated using PBE-D2 or vdW-DF (Table 1), and the *E*_ad_ values calculated using PBE-D2 and vdW-DF are more similar. This is because the PBE functional does not lead to the correct –*C*_6_/*R*^6^ dependence of the dispersion interaction energy on the range of intermolecular distance *R*^39^. From the comparison between *E*_ad_ calculated using PBE and *E*_ad_ calculated using PBE-D2/vdW-DF, it can be inferred that the dispersion interactions take up a large portion in the adsorption energy. This is also found in the adsorption of some other organic molecules on GP both by theoretical calculation and experiment^40^.

Aside from the dispersion interactions, electrostatic polarization is another source of the adsorption energy. For example, in the most stable configuration of GD-Gly (Fig. 1(f)), the N atom and the four atoms of –COOH of Gly molecule are approximately located in a plane which is parallel to GD, and the Gly molecule lies near the vertex of a large C-ring of GD. In the Gly molecule, the electronic density near the O and N atoms is larger due to their strong electronegativity. So, the GD surface will be polarized by the O and N atoms. For the most stable GD-Glu, GD-His and GD-Phe (Fig. 1(f)), we also found that the -COOH and –NH_2_ groups of AAs tend to be close and parallel to the GD surface. Furthermore, the acetylenic bonds in GD have more π-electrons than the ethylenic bonds in GP and thus cause stronger electrostatic polarization. Additionally, in GD-His/GD-Phe the aromatic ring of His/Phe gets close to the hexagonal ring of GD (see Fig. 1(f)) due to the π-π interactions^38^,^41^.

### Energy bands

**Figure 2:**
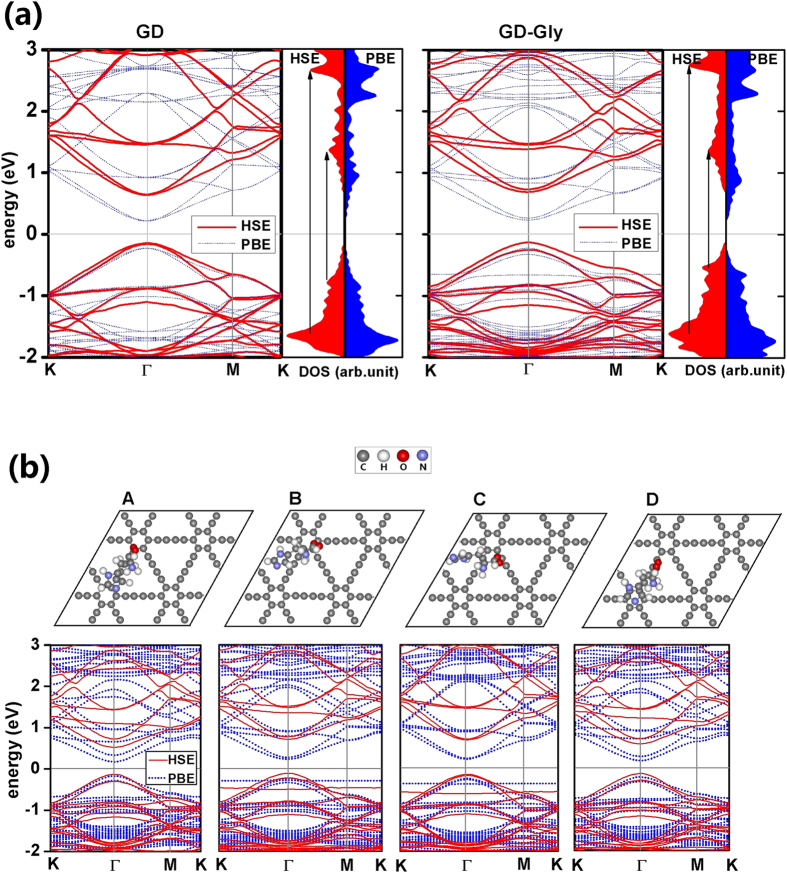
(a) The energy bands and DOS of GD and GD-Gly with (2 × 2) GD supercell at the PBE/HSE06 level. Some optical transitions are indicated by arrows. The Fermi energy is set to zero. (**b**) 4 instant configurations of GD-His and their energy bands at the PBE/HSE06 level.

To clarify the effect of AA molecules on the electronic properties of GD, the energy bands and the density of state (DOS) of (2 × 2) GD supercell are plotted with and without AA molecules. For the GD-AA systems, the band gap calculations were performed for the most stable configurations. The results calculated using the PBE functional and the Heyd-Scuseria-Ernzerhof (HSE06) hybrid functional^42^,^43^ are listed in Table 2. For pure GD, the band gap is *E*_g_ = 0.44/0.83 eV at the PBE/HSE06 level, respectively. Our PBE value is close to the value *E*_g_ = 0.46 eV in ref. 31. A direct band gap locates at the Γ-point (the left panel of Fig. 2(a)). Some optical transitions between the peak values of DOS (shown by arrows in Fig. 2(a)) cause the photon absorption peaks (see the Section “**Optical properties**”). For GD-Gly, the energy band profile and DOS are plotted in the right panel of Fig. 2(a). The extra occupied states in the energy band profile are molecular energy levels of Gly. The PBE/HSE06 functional gives a band gap *E*_g_ = 0.46/0.87 eV, respectively. For GD-Glu, GD-His and GD-Phe, the PBE/HSE06 functional gives *E*_g_ = 0.50/0.94, 0.40/0.77 and 0.48/0.90 eV, each respectively. In the above results, all of *E*_g_ calculated using the HSE06 functional are larger than their corresponding PBE values.

Next, the change of *E*_g_ induced by the motions of AA molecules at room temperature is displayed. In the MD simulations, some instant configurations of GD-AA systems are selected and relaxed by the geometry optimization, and then the energy bands are calculated. As an example, 4 instant configurations of GD-His are chosen and optimized as **A**, **B**, **C** and **D**, with their corresponding energy bands shown in Fig. 2(b). In the migration of His, the band gap *E*_g_ ranges from 0.34~0.51/0.70~0.89 eV at the PBE/HSE06 level, respectively.

### Optical properties

**Figure 3:**
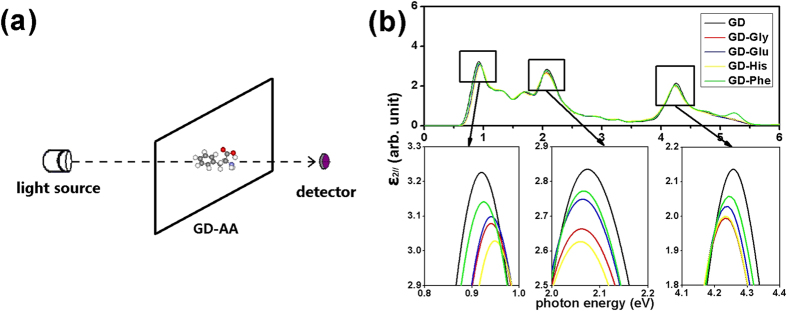
(a) The sketch of the absorption spectrum measurement for GD-AA. (b) The imaginary part of the dielectric function *ε*_2∥_ of pure GD, GD-Gly, GD-Glu, GD-His and GD-Phe.

In this section, the photon absorption spectra of GD with and without AA adsorption are investigated. For two-dimensional materials, the absorption spectrum detection is usually performed with the light beam perpendicular to the material (Fig. 3(a)), i.e. the photon polarization direction is parallel to the material. The calculated imaginary part of the dielectric function *ε*_2∥_ of pure GD, GD-Gly, GD-Glu, GD-His and GD-Phe are plotted in Fig. 3(b). For GD-AA systems, the calculations were all performed for the most stable configurations. For pure GD and GD-AA systems, the optical absorption spectra are characterized by three peaks around 0.9, 2.1, and 4.3 eV (Table 3). The first peak originates from the transitions around the band gap, and the other two result from the transitions between the peak values of DOS (shown by arrows in Fig. 2(a)). The shape of our optical spectra is close to ref. 44. According to the results (Fig. 3(b)), we see that the optical properties of GD-AA are different from the optical properties of pure GD. The AA molecules depress the three photon absorption peaks and make the first one blue-shifted and the other two red-shifted. The first absorption peaks of the GD-Gly, GD-Glu, GD-His and GD-Phe systems are located in the photon energy of 0.93~0.94 eV, and are depressed by 4.6%, 3.9%, 6.1% and 2.7%, respectively. The second absorption peaks of the GD-Gly, GD-Glu, GD-His and GD-Phe systems are located in the photon energy of 2.06~2.07 eV, and they are depressed by 6.1%, 2.5%, 7.5% and 2.3%, respectively. The third absorption peaks of the GD-Gly, GD-Glu, GD-His and GD-Phe systems are located in the photon energy of 4.23~4.24 eV, and are depressed by 6.7%, 5.1%, 6.5% and 3.8%, respectively. Such depressions of photon absorption peaks may be caused by the bending of GD induced by the AA molecules. For GD-Phe, the extra peak at 5.24 eV should be attributed to the characteristic absorption of the benzene ring. In summary, the obvious change in the photon absorption spectra of GD-AA systems should be used as a detection technique for AA molecules.

### Quantum electronic transport

**Figure 4:**
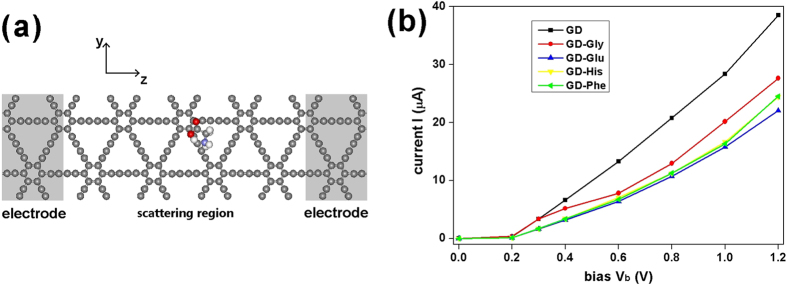
(a) The two-probe GD-Gly system for the quantum electronic transport simulation along the *z* direction. The two shaded areas are semi-infinite electrodes with periodic boundary conditions applied in the *y* and *z* direction. (**b**) The *I*-*V*_b_ curves of GD-AA systems and pure GD.

With the aim of designing GD-based devices applied within biology, medicine and pharmacy, such as biosensors, we investigate and compare the electronic quantum transport of the pure GD and GD-AA systems. Figure 4(a) shows the two-probe GD-Gly system used in the quantum transport simulations. The semi-infinite electrodes are modeled by the shaded areas in Fig. 4(a), with periodic boundary conditions applied in the *y* direction. The scattering region is modeled by four unit cells of electrodes with an AA molecule in the middle. For each AA molecule, the most stable adsorption configuration (mentioned in the above sections) was used in the simulation. According to the results, the current-bias curves for pure GD, GD-Gly, GD-Glu, GD-His, and GD-Phe (Fig. 4(b)) all show semiconductor-like feature with a turn-on voltage of 0.2 V. When the bias *V*_b_ is below 0.2 V, for either pure GD or GD with AA molecule the current *I* is near zero. When the bias *V*_b_ is above 0.2 V, the current *I* grows with increasing *V*_b_. At a same *V*_b_, the conductivity of the GD-AA systems is obviously lower than that of pure GD. At *V*_b_ = 1.2 V, the GD-Gly, GD-Glu, and pure-GD systems can be easily distinguished from each other by the current *I*, while the current of GD-His system gets close to that of GD-Phe. According to the above results, different kinds of AAs could be detected by the difference in current under a certain bias voltage. It implies that GD can be used for designing and fabricating biosensors.

## Discussion

We have shown the interactions between single-layer GD/GP sheet and Gly, Glu, His and Phe molecules. For each AA molecule, the adsorption energy on GD is larger than the adsorption energy on GP and the results indicate that the dispersion interactions take up a large portion in the adsorption energy. Furthermore, the strong electronegativity of O and N atoms in the AAs induce electrostatic polarization attractions with GD. At room temperature, the AA molecules constantly keep a distance of 2~3 Å from GD surface and randomly migrate and rotate on GD sheet. Such migrations and rotations cause the change of the GD bandgap. We found that in the presence of AAs, the characteristic photon absorption peaks of GD are depressed, with the first one blue-shifted and the other two red-shifted. It reveals that GD can be applied to detecting AAs through the absorption spectra. We also found that the current-bias behaviors of pure GD and GD-AA systems are distinctly different according to the quantum electronic transport simulations, which suggests GD as a promising biosensor material for the electronic detection of AAs.

AAs are the basic substances of life. The study of the interactions between GD and AAs is of great importance. Our results will provide guidance to future theoretical and experimental study. Overall, GD, with its unique character, would have a wide prospective application in biological devices or biomedicine.

## Methods

### Geometry optimization, MD simulation and adsorption energy

A (2 × 2)/(10 × 7) supercell is chosen for the single-layer GD/GP (Fig. 1(a)). The supercells were chosen to such a size so that the nearest distance between the replicas of adsorbed AA molecules is no less than 9 Å. The systems were simulated by a repeated slab model with a vacuum layer of 20 Å inserted in the perpendicular directions. *Ab initio* calculations were performed using the SIESTA code^45^. The improved Troullier-Martins norm-conserving pseudopotentials^46^ were employed. The PBE functional^32^, the PBE-D2 functional with dispersion correction^33^ and the non-local vdW-DF functional^34^,^47^ were applied. The grid mesh cutoff was set to 200 Ry, and double-ζ plus polarization (DZP) basis set was used. The Brillouin zone was sampled by using a (2 × 2 × 1) Γ-centered Monkhorst–Pack *k*-grid^48^. To test the used DZP basis set, geometry calculations were carried out for a single-layer GP sheet by using single-ζ plus polarization (SZP), DZP and triple-ζ plus polarization (TZP) basis set. By SZP/DZP/TZP basis sets, the lengths of C-C bonds were predicted to be 1.44/1.42/1.42 Å, respectively. So, DZP and TZP basis sets should have sufficient accuracy for GD/GP systems. To save computation time, we employed DZP basis set for the SIESTA code throughout the paper.

Geometry optimizations were performed using the PBE-D2 functional. The positions of all atoms were relaxed until the Hellman-Feynman force on each atom was less than 0.01 eV/Å. MD simulations were performed using the PBE-D2 functional. Verlet algorithm was employed with a time step of 1 fs. Temperature was controlled by Nosé thermostat. The MD simulations lasted for 5 ps. For AA on GD/GP, the adsorption energy *E*_ad_ is defined as


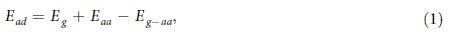


where *E*_g_, *E*_aa_ and *E*_g-aa_ denote the potential energy of GD/GP, isolated AA molecule and the GD-AA/GP-AA system, respectively. For all AAs, *E*_ad_ were calculated using the PBE/ PBE-D2/vdW-DF functional.

### Energy bands and photon absorption spectra

A (2 × 2) supercell is chosen for the single-layer GD (Fig. 1(a)). The calculations were performed using the projector-augmented wave method^49^,^50^ as implemented in the VASP package^51^,^52^. The PBE^32^ and HSE06^42^,^43^ functionals are employed. A plane-wave energy cutoff of 400 eV is used. The Brillouin zone is sampled by a 4 × 4 × 1 Γ-centered Monkhorst-Pack *k*-grid^48^. Gaussian smearing (ISMEAR=0 and SIGMA=0.05 for VASP) is used in the Brillioun zone integration. For plotting energy bands, 30 *k*-points were sampled along the high symmetry line of the Brillouin zone. To obtain smooth photon absorption spectra, the *k*-grid is increased to 6 × 6 × 1 in optical calculations. The photon polarization is parallel to the GD slab. The imaginary part of the dielectric function *ε*_2∥_(*ω*) was plotted for the photon energy *ħω* = 0~6 eV. 80 unoccupied bands are involved in the calculations.

### Quantum electronic transport

Quantum transport calculations were performed using non-equilibrium Green’s function method^53^ by the TRANSIESTA code^54^ implemented in SIESTA. The two-probe system is modeled by semi-infinite GD electrodes and a scattering region 4 times larger than the electrodes (Fig. 4(a)). Periodic boundary conditions were applied in the y directions. The improved Troullier-Martins norm-conserving pseudopotentials^46^ were employed. SZP basis set was applied. A 1 × 4 × 50 Monkhorst–Pack *k*-grid^48^ was used for electrodes. The electronic temperature was set to 300 K. For the bias voltage *V*_*b*_ applied on the *z* direction, the current *I* is given by Landauer-Büttiker formula^55^


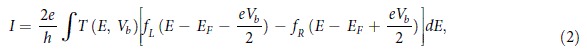


where *T(E, V*_*b*_) is the transmission, *E*_*F*_ is the Fermi energy of the electrodes, and *f*_*L*_ and *f*_*R*_ are the Fermi-Dirac distribution functions of both electrodes.

## Additional Information

**How to cite this article**: Chen, X. *et al*. Graphdiyne as a promising material for detecting amino acids. *Sci. Rep.* **5**, 16720; doi: 10.1038/srep16720 (2015).

## Figures and Tables

**Table 1:** The largest adsorption energy *E*_ad_ of Gly, Glu, His and Phe on GD/GP at the level of PBE/PBE-D2/vdW-DF functional.

**functional**	***E***_**ad**_ **on GD (eV)**			
	**GD-Gly**	**GD-Glu**	**GD-His**	**GD-Phe**
PBE	0.59	0.54	0.73	0.77
PBE-D2	0.90	0.90	1.22	1.27
vdW-DF	1.10	1.14	1.46	1.53
functional	*E*_ad_ on GP (eV)			
	GP-Gly	GP-Glu	GP-His	GP-Phe
PBE	0.23	0.43	0.45	0.56
PBE-D2	0.53	0.84	1.00	1.24
vdW-DF	0.54	0.94	1.14	1.37

**Table 2:** The band gaps *E*_g_ of GD, GD-Gly, GD-Glu, GD-His and GD-Phe at the PBE/HSE06 level.

**functional**	***E***_**g**_ **(eV)**				
	**pure GD**	**GD-Gly**	**GD-Glu**	**GD-His**	**GD-Phe**
PBE	0.44	0.46	0.50	0.40	0.48
HSE06	0.83	0.87	0.94	0.77	0.90

**Table 3:** The photon absorption peaks of pure GD, GD-Gly, GD-Glu, GD-His and GD-Phe.

**Photon energy (eV)**					
**peak**	**GD**	**GD-Gly**	**GD-Glu**	**GD-His**	**GD-Phe**
1^st^	0.92	0.94	0.94	0.95	0.93
2^nd^	2.07	2.06	2.06	2.06	2.07
3^rd^	4.26	4.23	4.24	4.23	4.24
